# A Mixed-Methods Approach to Investigating Social and Emotional Learning at Schools: Teachers’ Familiarity, Beliefs, Training, and Perceived School Culture

**DOI:** 10.3389/fpsyg.2021.518634

**Published:** 2021-06-01

**Authors:** Anja Schiepe-Tiska, Aiymbubu Dzhaparkulova, Lisa Ziernwald

**Affiliations:** Centre for International Student Assessment (ZIB) e.V., TUM School of Education, Technical University of Munich, Munich, Germany

**Keywords:** social and emotional learning (SEL), self-awareness, self-management, social-awareness, teachers’ attitudes, mixed-methods research

## Abstract

Education advocates argue that effective schools should foster multidimensional educational goals that not only include cognitive but also non-cognitive outcomes. One important non-cognitive outcome are social and emotional skills. Previous research showed that for enhancing students’ social and emotional learning (SEL) one of the most important factor is the teacher. Hence, the present study investigated teachers’ familiarity, beliefs, training, and perceived school culture with regard to social and emotional learning and its facets self-awareness, self-management, and social-awareness by applying a convergent parallel mixed-method design. We conducted in-depth interviews and an online survey with secondary teachers from different countries. The reason for collecting both qualitative and quantitative data was to obtain different but complementary data on the same topic in order to bring greater insight into this research question than would have been obtained by either type of data separately. Teachers reported an uncertainty and a lack of professional skills and knowledge in delivering SEL instructions that was particularly low for self-awareness and self-management. Therefore, in both study parts, teachers expressed strong interest in receiving professional SEL training. However, schools rarely provide resources (instruction materials, specific courses or activities) or create conditions (training teachers, devoting teaching hours, increasing number of counselors at schools, receiving school administration support), that would promote teachers’ instruction of SEL. The results do not only add to researchers’ knowledge about teachers’ SEL familiarity, beliefs, training, and school culture, but are also relevant for policymakers, administrators, and school staff by identifying critical aspects that prevent successful SEL in schools.

## Introduction

Since educational institutions have been established, ongoing discussions about the objectives of schooling have emerged. Schools ensure that students gain skills in reading, writing, math, and science. They also promote a good comprehension of history, literature, arts, foreign languages, and diverse cultures (Greenberg et al., 2003). These knowledge and skills are undeniably important cognitive student outcomes. However, over the past decade, the attention of which outcomes students shall achieve broadened from these cognitive to so-called “non-cognitive” factors as additional important school outcomes (e.g., Rieger et al., 2017) and concepts of educating the “whole child” became more prominent (Liew and McTigue, 2010). According to multiple reviews and studies, non-cognitive factors are essential for success in education as well as in occupation (e.g., Kautz et al., 2014; Rieger et al., 2017). They are characterized as constructs that are not identified with traditional indicators of cognitive capability or intellectual functioning (Rieger et al., 2017) and are often described under such terms as socio-emotional skills, character, personality, or 21st-century skills.

One important non-cognitive facet is social and emotional learning (SEL), including, among other things, students’ self-awareness, self-management, and social awareness (Durlak et al., 2011; OECD, 2015). These skills foster learners’ performance (e.g., Corcoran et al., 2018) and facilitate positive social behaviors, goal orientations, emotion management, and social relationship-building skills (Elias and Arnold, 2006; OECD, 2015). Moreover, they reduce behavior problems and psychological distress (Harrell et al., 2009; Durlak et al., 2011; Sklad et al., 2012; Wigelsworth et al., 2016; Taylor et al., 2017). Hence, they are important skills that help students succeed in school, work, personal life, relationships with families and friends, and society in general (cf. Mahoney et al., 2018).

Previous studies on SEL in the school context mostly confirmed their positive effects across all grade levels (e.g., Harrell et al., 2009; Durlak et al., 2011; Sklad et al., 2012; Wigelsworth et al., 2016; Taylor et al., 2017; Corcoran et al., 2018). However, among the critical factors that influenced an effective SEL program implementation, teachers themselves were among the most crucial features (Graczyk et al., 2006; Durlak et al., 2011). Their attitudes and beliefs about SEL affected the adoption, outcome, and sustainability of SEL programs (Gingiss et al., 1994; Parcel et al., 1995; Bowden et al., 2003; Zinsser et al., 2014).

Although teachers’ importance had been acknowledged early, thus far, only few qualitative and quantitative studies have examined teachers’ perceptions of SEL. If so, they focused mostly on preschool and elementary school teachers (Durlak et al., 2010, 2011; Wigelsworth et al., 2016; Taylor et al., 2017). Moreover, all of the mentioned studies examined teachers’ understanding of SEL in general but did not systematically target specific SEL facets. Besides the integration of structured evidence-based SEL programs, only a few studies explored schools’ and teachers’ own attempts, initiatives, and instructional practices to enhance students’ social and emotional competencies (Zinsser et al., 2014).

The present study adds to this research gap and aims at investigating secondary school teachers’ SEL familiarity, beliefs, training, and perceived school culture. In addition, the study applies a mixed-methods design, extending prior research by combining the collection of qualitative and quantitative data in order to get a more complete and nuanced picture than would have been obtained by either approach separately. This is not only valuable for researchers by enhancing their knowledge about teachers’ SEL familiarity, beliefs, training, and perceived school culture. It is also important for policymakers, administrators, and school staff by identifying critical aspects that prevent successful SEL in schools.

### The Concept of Social and Emotional Learning

Social and emotional learning involves processes of thinking, feeling, and behaving in order to become aware of the self and others, to regulate self-behavior and the behavior of others, and to make responsible decisions (Elias et al., 1997; Brackett and Rivers, 2014). Five interrelated core social and emotional competencies are defined: (1) self-awareness, (2) social awareness, (3) self-management, (4) relationship skills, and (5) responsible decision-making (Yopp et al., 2017). The present paper focuses on the first three competencies—self-awareness, social awareness, and self-management. These facets are less often and less explicitly addressed in teaching than relationship skills and responsible decision-making (Beland, 2007). Moreover, they can be more clearly distinguished while relationship skills and responsible decision-making are already at the intersection of a number of other SEL components (Denham and Brown, 2010).

*Self-awareness* is characterized as the ability to carefully identify one’s emotions, thoughts, interests, and values, as well as to understand how these impact one’s behavior (Eklund et al., 2018). In addition, it involves the ability to evaluate one’s strengths and limitations accurately and maintain a well-grounded sense of self-efficacy and sense of self-confidence (Denham and Brown, 2010; Brackett and Rivers, 2014).

*Self-management* involves self-discipline, motivation, goal setting, and stress management (Dusenbury et al., 2011). It is the ability to regulate one’s emotions, thoughts, and behaviors in various situations, and be able to set and monitor progress toward personal and academic aims (Brackett and Rivers, 2014; Eklund et al., 2018). Thus, it shares some similarities with the concept of self-regulated learning (Schunk and Zimmerman, 2012).

*Social awareness* is defined as having respect and empathy for others and understanding others’ perspectives and feelings (Zins and Elias, 2007; Denham and Brown, 2010). It is also the ability to perceive similarities and differences among people (Denham and Brown, 2010).

These competencies develop at different age levels, and most structured SEL intervention programs focus on preschool or elementary school children (Durlak et al., 2010, 2011; Wigelsworth et al., 2016; Taylor et al., 2017). However, early adolescence is also an important stage to enhance SEL as the social brain changes and reorganizes structurally and functionally (Blakemore and Mills, 2014). It is a period of intensive learning, exploring, and taking new opportunities, along with facing possible health and behavioral challenges, which can continue into adulthood (Yeager, 2017). Hence, school and teaching can still influence students’ social and emotional skills even at these later stages of age.

### Teachers’ Social and Emotional Learning Familiarity, Beliefs, Training, and Perceived School Culture

To facilitate students’ SEL, teachers need to be familiar as well as feel comfortable, committed, and trained in teaching social and emotional competencies. Moreover, the match with the culture of the school they are employed at can affect their SEL teaching practices (cf. Brackett et al., 2012).

Previous qualitative studies gave first hints that teachers seem to be not very familiar with the concept of SEL and that their knowledge is limited. For example, Esen-Aygun and Sahin-Taskin (2017) interviewed Turkish elementary school teachers and reported that most teachers had not heard about the concept of SEL. However, although they were not familiar with the concept, they did provide some activities to develop social and emotional skills when problems in the classroom came up and emphasized the importance of developing social and emotional competencies. Likewise, Triliva and Poulou (2006) interviewed Greek elementary school teachers and reported low levels of familiarity.

Beliefs indicate teachers’ perceptions and judgments. They strongly influence teachers’ filter of information, the framing of a situation, and guide their intentions. Hence, beliefs affect teachers’ teaching practices and experiences (Pajares, 1992; Fives and Buehl, 2012; Trivette et al., 2012). Two important SEL beliefs are teachers’ *comfort with and confidence in teaching SEL* as well as their *commitment to improve their own skills in teaching SEL* (Brackett et al., 2012).

While quantitative research often reports medium levels of teachers’ SEL comfort (e.g., Collie et al., 2011, 2012, 2015; Brackett et al., 2012; Poulou, 2017a), more in-depth qualitative studies revealed that teachers report uncertainty in teaching SEL. For example, Buchanan et al. (2009) found that in their sample of United States kindergarten through eighth-grade teachers, only a few felt confident in teaching SEL (22%), although half of them already participated in an SEL program. Hence, quantitative and qualitative studies revealed inconsistent findings about teachers’ comfort in teaching SEL.

When participating in structured SEL programs, teachers’ comfort and confidence in their abilities are related to their SEL practices’ effectiveness, as they are more likely to continue using a program (Buchanan et al., 2009). Teachers’ comfort in teaching SEL predicts higher teaching commitment in general (Collie et al., 2011) and is related to higher levels of self-efficacy and job satisfaction (Collie et al., 2012). In addition, high levels of comfort with implementing SEL practices are related to close and supportive teacher–student relationships in elementary school (Poulou, 2017a). Zinsser et al. (2014) showed that high supportive preschool teachers were more confident in using SEL strategies than medium supportive teachers. They used more often interactional SEL practices through modeling, coaching, or scaffolding childrens’ emotional experiences. A prescribed SEL curriculum was only used secondary to their interactions. In contrast, medium supportive teachers relied heavily on prescribed curricula during predefined times of the day.

An important aspect that is related to teachers’ confidence and self-efficacy with providing SEL instructions is teacher training and qualification (Zins et al., 2004; Buchanan et al., 2009; Durlak, 2016). Although particularly elementary school teachers are interested in and committed to learn about how to develop SEL (Collie et al., 2011; 2015; Esen-Aygun and Sahin-Taskin, 2017; Poulou, 2017a), most studies have shown that neither pre-service nor in-service teachers receive training in teaching SEL (Jones and Bouffard, 2012; Schonert-Reichl and Zakrzewski, 2014) or in developing their own SEL competencies (Jennings and Greenberg, 2009; Oberle and Schonert-Reichl, 2017) outside of the participation in structured SEL programs. As teachers at the secondary school level are asked even less explicitly to teach SEL, training and qualification are also rather scarce (see also Oberle and Schonert-Reichl, 2017). A content analysis of required courses in teacher preparation programs in the United States revealed that only a few programs offered SEL course content (between 1% and 13% of almost 4,000 courses in 300 colleges of education; Schonert-Reichl et al., 2016).

In addition to person-centered explanations for why SEL programming promotes positive outcomes, findings indicate that it is also important to consider systemic and environmental factors (Greenberg et al., 2003). Programs that occur in classrooms or throughout the school are likely to be impacted by these environments’ organizational and ecological features. A few prevention and promotion studies have begun to explore the importance of classroom, school, and neighborhood contexts on program outcomes to illustrate how a broader ecological perspective can enhance the understanding of program effects (Tolan et al., 1995; Aber et al., 1998; Metropolitan Area Child Study and Research Group, 2002; Boxer et al., 2005). When the perceived school culture matches the individual teacher’s beliefs, he or she reports lower stress and greater job satisfaction (Skaalvik and Skaalvik, 2011). In elementary schools that value SEL by supporting and promoting SEL teaching, teachers were more committed to their school and teaching in general (Collie et al., 2011). In addition, high levels of elementary school principals’ support are positively related—and needed—to implement SEL teaching practices effectively (Wanless et al., 2013; Schonert-Reichl et al., 2015). Asking teachers about perceived barriers for teaching SEL, one particular barrier they report is the lack of classroom time.

### Differences Between Facets of Self-Awareness, Self-Management, and Social Awareness in Teachers’ Social and Emotional Learning Familiarity, Beliefs, Training, and Perceived School Culture

Thus far, single facets of SEL or comparisons of different facets have been investigated rarely. For teachers’ familiarity with SEL, Triliva and Poulou (2006) found that elementary school teachers were more familiar with the facet of social development as compared to emotional learning. Schonert-Reichl et al. (2016) conducted a content analysis of required courses in teacher preparation programs, and their results revealed that only 13% of the United States teacher preparation programs offered at least one course including information on relationship skills, 7% for responsible decision-making, 6% for self-management, 2% for social awareness, and approximately 1% for self-awareness. These results emphasize that training opportunities are overall scarce but that almost no offers exist for social and self-awareness. For the perceived school culture, thus far, no studies investigating differences between facets of SEL exist.

## Present Study

The current mixed-methods study examines teachers’ SEL familiarity, beliefs, training, and perceived school culture. Thus far, studies on this topic are limited and have only provided a partial view by using either a qualitative or a quantitative approach (see Zinsser et al., 2014, for an exception).

For our first research questions, we conducted semi-structured interviews in order to develop an in-depth understanding of how teachers describe SEL in general and its facets’ self-awareness, self-management, and social awareness in particular (RQ 1a). In addition, we were interested in exploring how comfortable and trained teachers feel for teaching SEL (RQ 1b). Based on previous research with preschool and elementary school teachers and the assumption that secondary school teachers are less explicitly asked to address SEL, we expected that secondary school teachers would not be very familiar with and trained in teaching SEL. Moreover, we wanted to describe how supportive teachers perceive their school culture for teaching SEL (RQ 1c).

A quantitative survey focused on differences between the three facets of SEL. We examined whether there were any differences in teachers’ reported self-awareness, self-management, and social awareness regarding teachers’ comfort, commitment, and school culture (RQ2). Based on the qualitative results of Triliva and Poulou (2006), who found that teachers were more familiar with the facet of social development compared to emotional learning, we assumed that teachers might report to be more comfortable in teaching social awareness compared to self-awareness and self-management. For teachers’ commitment toward learning about SEL, we expected high levels of commitment in general, as previous studies with elementary school teachers showed that they were highly committed to learn about how to teach SEL (Collie et al., 2011, 2015; Esen-Aygun and Sahin-Taskin, 2017; Poulou, 2017a). However, based on the finding that in teacher preparation programs, only a few offered SEL course content and, if so, they focused in particular on self-awareness and social awareness (Schonert-Reichl et al., 2016), we expected that teachers’ reported commitment in learning about self- and social awareness would be higher as compared to their commitment in learning about self-management. As, thus far, no other studies have compared different facets of SEL, we did not specify any further hypotheses.

In addition, we investigated to what extent the interview results on familiarity, comfort, training, and perceived school culture agreed with the quantitative results on secondary school teachers’ beliefs about the specific facets self-awareness, self-management, and social awareness (RQ3). Previous research using either qualitative or quantitative methods already points out that differences in the general level of teachers’ comfort in teaching SEL exist (e.g., Triliva and Poulou, 2006; Buchanan et al., 2009; Collie et al., 2011, 2012, 2015; Brackett et al., 2012; Poulou, 2017a). However, overall, there is a need for a more complete understanding through comparing and synthesizing both personal experiences of teachers investigated with interviews that allow a thorough examination about SEL in general (i.e., qualitative data) and gaining more standardized results (i.e., quantitative data) about different facets of SEL.

## Materials and Methods

### Study Design

**FIGURE 1:**
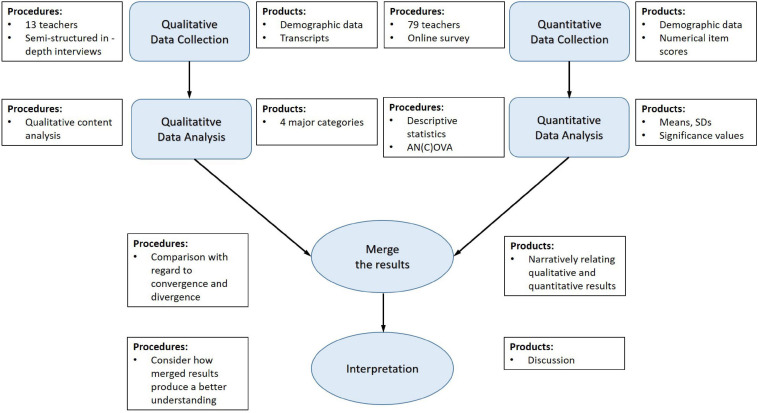
Convergent parallel mixed-methods study design.

The present study used a mixed-methods design. Mixed-methods research collects, analyzes, and mixes both quantitative and qualitative data in a single study (Creswell and Plano Clark, 2018). A convergent parallel design was applied; that means qualitative and quantitative data were collected in parallel, analyzed separately, and then merged. For the qualitative part, semi-structured in-depth interviews with secondary school teachers were conducted. Interviews have the advantage that teachers had more space to answer questions more openly and elaborately. Moreover, their individual needs and ideas could be better addressed and their context and everyday setting could be better taken into account. For the quantitative part, an online-based survey was set up. This has the advantage that an established, standardized, valid questionnaire could be adapted and used (Brackett et al., 2012) in order to compare teachers’ reported comfort, commitment, and schools’ culture between the three facets self-awareness, self-management, and social awareness. The integration involved merging the results from the qualitative and quantitative data so that a comparison could be made and a more complete understanding emerges than that provided by the quantitative or qualitative results alone (Heyvaert et al., 2013; Creswell and Plano Clark, 2018). Figure 1 shows an overview of our study design.

### Participants and Procedures

#### Qualitative Part

For the recruitment of interview participants, a purposive sampling strategy was used that enables researchers to select respondents based on specific criteria (Etikan et al., 2016). Most of the teachers were targeted based on the criterion of having experience in teaching in secondary schools with a particular emphasis on ninth-grade students. Overall, 14 respondents agreed to participate in the study. Ten of them were enrolled in a master’s program on “Research on Teaching and Learning” and were classmates of the second author, who conducted the interviews in this study. Four respondents were working as full-time teachers in Kyrgyzstan and were former classmates and colleagues of the interviewer. None of them had participated in a structured SEL program yet. Research participants were invited to take part in the interview through face-to-face recruitment.

Table 1 shows the characteristics of our study participants (see also Supplementary Appendix A for a detailed description of the interview participants). One teacher (#8) had experience in teaching preschool students only. Hence, in order to better compare and interpret our results, we excluded this teacher from the following analyses. In sum, 13 interviews were analyzed.

**TABLE 1:** Description of the qualitative and quantitative sample.

		**Interview**		**Questionnaire**	
		**Frequencies**	***M* (*SD*)**	**Frequencies**	***M* (*SD*)**
Gender	Female	10		73	
	Male	3		6	
Subjects taught	Science and Math	3		26	
	Social Science	3		10	
	Sports	0		4	
	Languages	6		34	
	Arts	1		5	
Type of school	Private	2		11	
	Public	9		56	
	Public and private	2		12	
Country of teaching	Asia	2		12	
	Europe	4		20	
	Kyrgyzstan	4		24	
	United States	3		19	
	Others	0		4	
Grades taught	Elementary	1		1	
	Secondary	12		78	
Age			27.5 (6.8)		34.7 (11.1)
Years of experience			4.9 (4.2)		9.7 (9.4)

*Total of interview respondents N = 13. Total of questionnaire respondents N = 79.*

Interviews lasted between 20 and 60 min, with an average interview time of 30 min. Most interviews were conducted face-to-face with single teachers. One interview was conducted online *via* Skype and one through a telephone call. A trained qualitative researcher with a bachelor’s degree in sociology from the American University of Central Asia held all interviews (i.e., second author). Prior to the data collection of the current study, the researcher had three years of experience in conducting qualitative data collection and analysis. Participants who were enrolled in the master’s program were interviewed in English, as this was the official language of the master’s program. Three teachers were interviewed in Russian and one in Kyrgyz, which were the mother tongues of the interviewer and the participants.

Research participation was confidential and on a voluntary basis. All interviews were recorded with respondents’ permission asked at the beginning of each interview (see Appendix B). The study was conducted according to the *Ethical Principles of Psychologists and Code of Conduct* of the American Psychological Association, 2019. An ethics approval was not required by institutional guidelines or national regulations in line with the “*German Research Foundation*” guidelines, as the used data were anonymized, and no disclosure outside the research is possible.

#### Quantitative Part

Initially, 88 respondents were recruited through the researcher’s network and social media platforms, such as Facebook and Instagram. Again, they were recruited based on the criteria of having teaching experience at secondary schools. Interview participants were also asked to participate in the questionnaire. Nine participants did not complete the survey and thus were excluded from the data collection process. Overall, 79 respondents participated. Table 1 shows a description of the sample.

An online survey was created using Google forms^1^. Google forms is compliant with the European General Data Protection Regulation (Google, 2020), and participants were treated in accordance with the American Psychological Association’s Ethics Code. First, they were informed about the study aims: (1) to examine how teachers and schools support students’ social and emotional learning in terms of students’ self-awareness, self-management, and social awareness skills and (2) to compare perceptions of teachers and students regarding opportunities that schools and teachers provide to students for learning self-awareness, self-management, and social awareness skills. In addition, they were informed that participation in this study is conducted voluntarily. All data are confidential and will be used only in the frames of this research.

### Research Instruments

#### Qualitative Part

An interview guide with 20 questions was developed (see Appendix B). The guide had four sections. The first section consisted of introductory and background questions as well as general questions about the definition of SEL and its facets self-awareness, self-management, and social awareness. In this section, after introducing themselves, teachers were asked to explain their own understanding of the terms SEL, self-awareness, social awareness, and self-management. After that, a definition of these concepts was provided to the interviewees in order to have a common understanding when discussing the following questions. The second section aimed at exploring how schools support students’ SEL. The third and fourth sections focused on how comfortable and trained teachers felt in teaching SEL and how they individually supported SEL in their classrooms. Some of the questions were adapted from the interview study by Esen-Aygun and Sahin-Taskin (2017). In addition, demographic questions were asked (see Appendix B for the full list of questions asked in the qualitative part).

During the interviews, all questions had been asked. However, the order of question emerged from the course of conversation. The interview guide was translated into Kyrgyz and Russian languages. The translation quality was tested with three researchers, who translated the interview guide from English to Russian and Kyrgyz and *vice versa*. After piloting the interview guide with four teachers, it was adjusted by reformulating some items that were initially conceptualized as “personality development” to “SEL” and its specific items.

#### Quantitative Part

Teachers’ comfort with teaching SEL, their commitment to learn about SEL, and their perception about whether their school culture supports SEL were assessed using an adaptation of the established teachers’ SEL beliefs scale (Brackett et al., 2012). As the original questionnaire does not distinguish between different SEL facets, we adapted the questionnaire by presenting a definition of the corresponding facet followed by the items of the original scale (see Appendix C for the full list of constructs that have been assessed in the quantitative part). We substituted the term “social and emotional learning” from the original items with the corresponding facet. Comfort, commitment, and perceived school culture were assessed with four items concerning teachers’ self-awareness, self-management, and social awareness. Therefore, the final scale consisted of 36 items (12 per facet). Cronbach’s alpha revealed good to high internal consistencies (Table 2). Teachers rated their agreement with each item on a 5-point Likert-type scale (from strongly disagree to strongly agree).

**TABLE 2:** Descriptive statistics and Cronbach’s alpha for the subscales.

	**Comfort**					**Commitment**					**Perceived school culture**				
	***M***	***SD***	**Min**	**Max**	**Cronbach’s alpha**	***M***	***SD***	**Min**	**Max**	**Cronbach’s alpha**	***M***	***SD***	**Min**	**Max**	**Cronbach’s alpha**
Self-awareness	3.15	0.77	1.75	5.00	0.87	4.36	0.57	2.50	5.00	0.84	2.56	0.94	1.00	5.00	0.89
Self-management	3.35	0.74	2.00	5.00	0.88	4.34	0.47	3.25	5.00	0.79	2.83	1.01	1.00	5.00	0.93
Social awareness	3.97	0.63	2.25	5.00	0.90	4.35	0.46	3.00	5.00	0.76	3.73	0.87	1.25	5.00	0.94

### Analyses

#### Qualitative Part

The same researcher who had conducted the interviews also transcribed and analyzed the recorded interviews. The interviews were anonymized and transcribed verbatim. We used an iterative process of deductive and inductive qualitative content analysis (Cole, 1988). Qualitative content analysis aims to acquire a condensed and comprehensive explanation of the phenomenon. It results in concepts or groups representing the phenomenon (Elo and Kyngäs, 2008). Following the process described by Elo and Kyngäs (2008), there were three phases: preparation, organizing, and reporting. In the preparation phase, we selected the 13 transcribed interviews as units of analysis. We decided to focus on the manifest content only. Latent contents, for example, sighs and laughter, were not analyzed, as they were not considered relevant for our research questions. In order to get familiar with the data, the transcribed protocols had been read through several times.

For organizing our material, we developed a structured categorization matrix according to our main research questions. We defined four categories for coding teachers’ responses. The first category, “Definition of and familiarity with SEL,” was developed based on prior interview studies showing that teachers were not very familiar with the general concept of SEL (Triliva and Poulou, 2006; Esen-Aygun and Sahin-Taskin, 2017). The other three coding categories, “SEL instruction comfort,” “SEL experience and training,” and “SEL school culture,” reflect similar categories defined by Brackett et al. (2012), which was also the theoretical foundation for the questionnaire used in the quantitative part. One main difference is that instead of asking how committed teachers felt for attending a training, as it has been done in the questionnaire by Brackett et al. (2012), we explicitly included questions regarding actual training, which teachers may have received in SEL or teaching SEL. For coding, first, we chose aspects from the data that fitted our predefined categorization frame. Second, we considered (and coded) themes that occurred from multiple interviews, which had not been predefined, such as “stating the importance of SEL,” “commitment for SEL training,” “worries and complaints related to uncertainties,” and “reasons for discomfort in teaching SEL.” Our goal was to collect a detailed description of the phenomenon and not to generate generalizability of the findings, although patterns and naturalistic generalizations emerged from the data (Creswell and Plano Clark, 2018).

#### Quantitative Part

Quantitative results from the questionnaire were analyzed using SPSS 26. We conducted a set of ANCOVAs with repeated measurement design. According to Field (2009), “repeated measures” is a term used when the same participants participate in all conditions of a study. In our study, conditions were the three SEL facets self-awareness, self-management, and social awareness (see also Gebauer and McElvany, 2017, for a similar approach). Hence, comfort, commitment, and perceived school culture were used as dependent variables and SEL facets as independent variables with three manifestations (self-awareness, self-management, social awareness). In addition, we controlled for teachers’ age, years of teaching experience, type of school, subjects taught, and country of origin. Besides, for the country of origin, the covariates were not significant. Hence, we further report only the results including the covariate when it showed a significant effect.

## Results

### Qualitative Results

#### Definition of and Familiarity With Social and Emotional Learning, Self-Awareness, Self-Management, and Social Awareness

In the present study, teachers were rather unsure whether they know the concepts and terms of SEL in general or the three facets in particular. Hence, they mostly described their own understanding of these concepts. When defining SEL, teachers mostly explained it as a concept that fosters social skills, such as building friendships and relationships, working in teams, along with emotional learning that promotes exploring your emotions and emotional states.

I would assume that it [SEL] would have to do with students’ ability to develop social skills with other students, peers, as well as adults. And then, emotional: I would assume that would be behavioral management and dealing with child’s ability to self-regulate, participate in the classroom, you know without misbehaving, things like that (Teacher #12, United States).

I think it is something with a pedagogical content, when you actually really say “OK, when you don’t really only teach something, but you really try to develop students as a person and their character and everything that involves within that.” So, I think it is much more about the person and their character building (Teacher #7, Germany).

While providing a general definition, teachers seemed to be intuitively aware of the three facets self-awareness, self-management, and social awareness without knowing and explicitly stating them.

Once teachers described their general understandings of SEL, they proceeded to provide definitions about the facets self-awareness, self-management, and social awareness.

*Self-awareness* was a concept that teachers reported to be most uncertain about how to describe it. In most cases, the term was conceptualized as “*knowing yourself*” and “*building personal identity*.”

Self-awareness is something really important. I think it is kind of being aware of what you are doing or why you are doing and being aware of yourself basically (Teacher #10, Turkey).

Self-awareness could be broad. You could even get into building self-identity, how you identify yourself in terms of culture, background anything like that (Teacher #12, United States).

Teachers related *self-management* mostly to skills of self-regulation and discipline. They defined this concept in relation to managing learning (behavior and school tasks) and managing lifelong goals (goal setting and regulation).

Self-management is about self-discipline, managing your own schedule, your own behavior, your learning; it must be about regulating yourself (Teacher #11, Turkey).

Self-management is all about goal achieving, how to separate their [students’] goals into small ones and also [connect goal setting] with their [students’] time management (Teacher #14, South Korea).

Teachers explained “*social awareness*” as a term that emphasizes students’ social skills such as relationship, friendship building, interacting with peers and other people, relating oneself to society, and being tolerant of people’s social diversity. Teachers pointed out that, to them, social awareness is an important skill that helps students adapt to society while being at school and also afterward in their adulthood.

Social awareness, in my understanding, is related to socialization process; it is when students learn how to interact with other people and adopt in new environments (Teacher #3, Kyrgyzstan).

Social awareness has to do not just with yourself, but also with others around you, and being aware that your actions may affect other people (Teacher #12, Finland).

In sum, teachers in the present study described the concepts from their personal understanding rather than from professional teacher education or training. They reported that they were not much aware of the terms, which made them feel uncertain in their responses. For social awareness in particular, teachers had a more broad definition in mind that also included aspects of the SEL facet relationship skills. Nevertheless, teachers explicitly pointed out the importance of SEL and personality development for students’ lifelong learning, life satisfaction, and success in school and also later in their career and relationship building.

#### Teachers’ Comfort and Training in Teaching Social and Emotional Learning

Teachers reported that they were not very comfortable and confident when they had to interact with students concerning their social and emotional education or needs. Their uncertainties were mostly related to worries and complaints about not having enough time for delivering instruction on SEL besides the content of the subject taught as well as a lack of materials and professional training regarding SEL.

You know we have limited time, we have certain content to cover, we have many students, all of that does not allow me to pay attention to every individual student’s interests, social and emotional needs. Because I do not work on that side of teaching a lot, I will be honest I cannot say I am confident or feel comfortable when it comes to emotions of students (Teacher #11, Turkey).

According to most interviewed teachers, their bachelor’s or master’s programs did not offer specific courses related to teaching SEL. Some teachers had classes on educational or pedagogical psychology on the topic of classroom management or dealing with behavioral problems. However, these classes focused more on intervention rather than prevention. Nevertheless, teachers mentioned that most of their skills and knowledge come from their daily teaching experience rather than from professional training.

Yes, we had courses on psychology or pedagogy, but I cannot say that I learned a lot from those courses. In fact, most of my experience on pedagogy comes from actual practical experience of teaching in the classroom. And definitely, there was nothing about teaching students to know about themselves, their interests, strengths and weaknesses, emotions or social skills. No, we did not study that (Teacher #11, Turkey).

I cannot remember such courses at university; I would say no, we did not study social and emotional education. And later at work, we did not receive training on that, we had some teacher conferences on how to work with kids with behavioral problems maybe that can relate a bit, we were discussing how to manage class when someone is disturbing lessons, but other than that, I cannot remember (Teacher #4, Kyrgyzstan).

Despite the fact that teachers in the present study mentioned a lack of educational and professional training on delivering SEL competencies, they have expressed their commitment to teach SEL competencies by relating to other trainings they got, as well as by trying to incorporate some information related to SEL through the means of their teaching methods, in-class activities, discussions, and personal conversations.

I taught in the urban setting for students coming from low economic background. She [student] was dealing with a lot at home and she was always acting up in the classroom and disrupting the classroom. And, so I think one of the things I helped her with was just again coping mechanisms—dealing with stress at home, learning to find her ways to regulate and calm down. This is something I learned in college. I was taught how to mediate between people and one of the things was, I think, self-regulating—learning to cope. I just taught her some things dealing with stress and I think it helped her a little bit. That is something you can use for everyday life, when you experience stress, you just find your own ways [of coping]. She did not want to participate, disrupt the class and yes we sat down after [class] and we spoke for 30 min and she was just telling me about everything at home (Teacher #12, United States).

In addition, interviewees highlighted that they would be interested in getting professional training about teaching SEL in general but were also interested in training about developing their own SEL skills.

Social and emotional skills have to be taught almost like a hard skill. You know what I mean, it is a sensitive topic, there can be sensitive issues. We [teachers] are not trained for that, we might have some pedagogical knowledge, like how to manage class, but it is not enough. In order to be comfortable and confident in knowing students’ emotions, something like emotional intelligence, in order to see if students know themselves well, we [teachers] need to understand ourselves how to figure that out first (Teacher #12, United States).

To summarize, teachers’ reported discomfort with teaching SEL was mostly related to the lack of professional training, materials, and time during lessons. Nevertheless, they stated high interest in receiving such trainings not only for teaching SEL but also for developing these skills for themselves.

#### Teachers’ Social and Emotional Learning Instruction and Their Perceived School Culture

Teachers, who worked in public schools, reported that they were not aware that SEL was part of their subjects’ curricula or study plans. They mainly argued that they have specific plans of covering required content information and achieving their learning objectives, which rarely relate to SEL. However, although not part of curricula or study plans, some teachers pointed out that they tried to incorporate aspects of self-awareness or self-management skills into their teaching through the reflection and discussion of the content, personal initiatives of discussing these terms with the class, or in personal conversations with students individually.

We watched so many videos and did many discussions afterward. I think my class was very different from other classes because I always bombed them with questions “Who are you?,” “Why are you here?,” “What do you do here?,” and they would really question and leave the class with thoughts, they really criticized [school] administration. I felt a little guilty, but for me it was important because in university where I studied we were taught critical thinking and I could find my true self through this. So, I wanted my students also to think who they are and what they believe in (Teacher #1, Kyrgyzstan).

Interviewees, who had experience in working at private schools, explained that their schools particularly emphasized developing students’ SEL by providing a variety of extracurricular activities such as arts, sports, or debating clubs. Teachers in Kyrgyzstan, for example, mentioned that presenting a wide range of extracurricular activities was also a “marketing strategy” of these schools in order to attract more students.

In a private school in order to attract clients so that their children are developing not only in terms of knowledge, but also in terms of personality development [schools had extracurricular activities]. For instance, in our school we have state standards according to which we should teach content knowledge. But we also try to develop different skills. For instance, we have drawing clubs and exhibitions. This year we had an art exhibition at the state museum of fine arts with students’ drawing and it makes students confident, it teaches them to express their thoughts (Teacher #2, Kyrgyzstan).

Teachers of public schools also reported extracurricular activities; for example, different types of sports, arts, or social activities, which aimed to foster different aspects of SEL.

At the schools, where I have worked, one of them did have these kinds of, I would call it, workshops, where you were able to do different things, which also included these social and emotional skills and learning and how to acknowledge them. But it did not come clearly like that, but behind something that people were doing, so for example, one of the schools had a cooking class and I would say the teacher took self-awareness, self-management, and social awareness in consideration while teaching (Teacher #13, Finland).

However, in the present study, some teachers explained that the variety of extracurricular activities sometimes means additional workload for them, particularly when they are responsible for these activities. Others raised worries that these activities might distract students from school content.

They have had so many choices of extracurricular activities that it was actually I think was too much for them. Well, for me it was a little too much workload on that because every teacher had to be responsible for at least two extracurricular activities (Teacher #6, China).

Most teachers reported that they share the perception that their schools do not emphasize and support SEL teaching at the school level.

One thing I think we [as a school] do not do a good job at is promoting students to find out who they are as a person and I know it takes time, right? I do not think schools do a good job at finding out what are ways to explore yourself (Teacher #5, United States).

I would honestly have to say no, we did not have outlets for students to learn these types of [SEL] skills or anything like that. Students come from low-income backgrounds, they deal with plenty of issues at home, at school or in the community and it [SEL] should go into the school, into classroom and there not many outlets for students to be aware of that [SEL] (Teacher #12, United States).

Moreover, from their perspective, schools’ focus is more on cognitive outcomes and managing the school and classes themselves as compared to SEL.

I cannot say that our school administrator was interested in promoting SEL. You know teachers already have many tasks, we need to deliver the knowledge, manage the class; we have only limited time and resources. The same with administration, they have many responsibilities with managing school, schedules, and different activities. I know SEL is important, but in practice, we just have too much work and SEL is, unfortunately, not very much a priority (Teacher #3, Kyrgyzstan).

You [teacher] have administration or policy that says “OK, by the end of this year these students need to know this, this, and this and if they don’t, it doesn’t look good for you.” What does it mean to yourself? Does it mean that scores are amazing and your teacher evaluation is great? Or is it more important for you to teach these students personal and social skills and grow them as a human being? (Teacher #5, United States).

In line with that, interviewees stated that they do not feel expected by schools to teach SEL skills unless students themselves show or address social or emotional needs.

One of my students in my class was having a terrible temper issue—it was anger issues. He could not control himself and he wanted to jump off [the roof]. At that time he was alarming the whole school and then the principal invited an educational psychologist and everybody had a closed door—indoor meeting. Nobody knows [about the meeting] and then I was inside there as well; we had to learn from that time what crisis is and how to respond to similar needs of students (Teacher #6, China).

However, several teachers in our study mentioned that they feel obligated and expected to respond to students’ social and emotional needs by students’ families and society in general.

Teachers are expected to be everything in the classroom. Especially in the States now there is a huge push [on teachers] by the society in general, teachers have to take on their role of being a mentor, helping students with emotional needs and things like that. I do not know maybe you have seen it in the news, bullying is a huge problem, we have students who are dealing with transgender roles, it is just a lot for a teacher. I think there is definitely an expectation placed on teachers to help students with those things. And it is not [assigned] by anyone in particular, it is not a requirement for schools to hire people with those skills, society is pushing that (Teacher #12, United States).

In sum, teachers have mentioned that in their school environment, cognitive and non-cognitive skills are interrelated. However, they felt that, in most cases, cognitive learning outcomes are more emphasized by schools or curricula. According to them, SEL is mostly incorporated by extracurricular activities or by teachers individually through teaching methods or student-teacher interactions. Hence, interviewees did not necessarily feel expected to teach SEL by their schools but reported a rather implicit expectation of families and society in general. In the present study, all teachers mentioned that their schools have at least one social worker or school counselor. However, they argued that this is not enough to respond to students’ social and emotional needs.

### Quantitative Results

The quantitative part examined the research question whether there were any differences in teachers’ reported self-awareness, self-management, and social awareness regarding their comfort, commitment, and school culture. Descriptive statistics and correlations are presented in Tables 2, 3. The means for the three facets for commitment are in general higher as compared to the means of comfort and school culture. In addition, teachers’ commitment is rarely related to comfort or perceived school culture across the different SEL facets, whereas teachers’ comfort shows mostly positive medium to high correlations with perceived school culture.

**TABLE 3:** Correlations.

		**Comfort**									**Commitment**									**Perceived school culture**					
		**Se-aw**			**S-man**			**So-aw**			**Se-aw**			**S-man**			**So-aw**			**Se-aw**			**S-man**		
		***r***	**CI 95%**	***p***	***r***	**CI 95%**	***p***	***R***	**CI 95%**	***p***	***r***	**CI 95%**	***p***	***r***	**CI 95%**	***p***	***r***	**CI 95%**	***p***	***r***	**CI 95%**	***P***	***r***	**CI 95%**	***p***
Comfort	Se-aw S-man So-aw	– **0.57 0.31**	[0.40, 0.70] [0.10, 0.50]	<0.001 0.005	– **– 0.47**	[0.28, 0.63]	<0.001	–																	
Commit ment	Se-aw	−**0.31**	[−0.50, −0.10]	<0.001	−0.17	[−0.38, 0.05]	0.129	0.14	[−0.08, 0.35]	0.216	–														
	S-man	−0**.22**	[−0.42, −0.00]	0.049	−0.17	[−0.38, 0.05]	0.124	−00.13	[−0.34, 0.09]	0.265	**0.40**	[0.20, 0.57]	<0.001	–											
	So-aw	0.15	[−0.07, 0.36]	0.196	0.01	[−0.21, 0.23]	0.918	−0.03	[−0.25, 0.19]	0.825	0.15	[−0.07, 0.36]	0.183	**0.50**	[0.31, 0.65]	<0.001	–								
Culture	Se-aw	**0.80**	[0.70, 0.87]	<0.001	**0.55**	[0.37, 0.69]	<0.001	**0.33**	[0.12, 0.51]	0.003	−**0.28**	[−0.47, −0.06]	0.013	−0.15	[−0.36, 0.07]	0.183	0.17	[−0.05, 0.38]	0.125	–					
	S-man	**0.40**	[0.20, 0.57]	<0.001	**0.69**	[0.55, 0.79]	<0.001	**0.37**	[0.16, 0.55]	0.001	−0.01	[−0.23, 0.21]	0.940	−0.04	[−0.26, 0.18]	0.749	0.12	[−0.10, 0.33]	0.308	**0.44**	[0.24, 0.60]	<0.001	–		
	So-aw	0.20	[−0.02, 0.40]	0.085	**0.33**	[0.12, 0.51]	0.003	**0.61**	[0.45, 0.73]	<0.001	0.07	[−0.15, 0.29]	0.558	−0.04	[−0.26, 0.18]	0.736	−0.06	[−0.28, 0.16]	0.600	0.18	[−0.04, 0.39]	0.111	**0.45**	[0.25, 0.61]	<0.001

*Se-aw, self-awareness; S-man, self-management; So-aw, social-awareness. Coefficients significant at the p < 0.05 level are in bold type. N = 79.*

The results of the ANCOVA with repeated measurement design for teachers’ comfort revealed a significant main effect for the three facets of SEL, *F*(2,148) = 30.71, *p* < 0.001, η^2^ = 0.29. In addition, a significant main effect was found for the covariate country of teaching *F*(4,74) = 3.03, *p* = 0.02, η^2^ = 0.14. United States teachers showed across all three facets higher levels of comfort as compared to teachers from other countries. However, the interaction between the three facets and country of teaching was not significant (*p* = 0.86). Pairwise comparisons revealed that teachers’ comfort with teaching social awareness was significantly higher than their comfort in teaching self-awareness [*M_Diff_* = 0.84, *SE* = 0.13, *p* < 0.001, 95% CI (0.53, 1.15)] and self-management [*M*_*Diff*_ = 0.67, *SE* = 0.13, *p* < 0.001, 95% CI (0.40, 0.93)]. No difference occurred in teachers’ comfort in teaching self-awareness and self-management [*M*_*Diff*_ = 0.17, *SE* = 0.11, *p* = 0.31, 95% CI (−0.83, 0.43)].

For teachers’ commitment to learn about SEL, no covariate was significant. When conducting an ANOVA with repeated measurement, Mauchly’s test indicated that the assumption of sphericity had been violated, χ^2^ = 14.52, *p* < 0.001. Therefore, the degrees of freedom were corrected using Greenhouse-Geisser estimates of sphericity. Teachers’ commitment did not significantly differ between the three facets of SEL, *F*(1.71,133.12) = 0.03, *p* = 0.95.

Teachers’ perceived supportive school culture differed between the three facets of SEL, *F*(2,156) = 52.62, *p* < 0.001, η^2^ = 0.40. The covariates did not reach significance. Pairwise comparisons showed that teachers’ perceived school culture with regard to social awareness was significantly higher as compared to their perceived school culture in teaching self-awareness [*M*_*Diff*_ = 1.17, *SE* = 0.13, *p* < 0.001, 95% CI (0.85, 1.49)] and self-management [*M*_*Diff*_ = 0.90, *SE* = 0.11, *p* < 0.001, 95% CI (0.62, 1.17)]. Their perceived supportive school culture in self-awareness and self-management did not differ significantly [*M*_*Diff*_ = 0.28, *SE* = 0.12, *p* = 0.06, 95% CI (−0.01, 0.56)].

### Mixed Methods

After analyzing the quantitative and qualitative data separately, the results from each were compared at the point of interpretation in order to identify similarities and differences. Convergent data analysis revealed that teachers seem to feel most familiar and comfortable in teaching the facet social awareness compared to self-awareness and self-management. Furthermore, for teachers’ training in SEL, the data confirmed each other. The interviewed teachers reported that they did not receive any SEL training but were highly interested in and committed to receive professional training in teaching SEL. These high levels of commitment were also reflected in the high scoring of commitment for the separate facets. Concerning school culture, the datasets partially confirmed and complemented each other. The finding that teachers reported that their schools and principals did not emphasize teaching SEL matches the low and medium ratings of school culture for the facets of self-awareness and self-management. However, for social awareness, quantitative and qualitative data diverged as teachers in the survey reported a high emphasis on fostering social awareness at the school level. Moreover, datasets were dissimilar in the level of comfort teachers reported with teaching SEL. Interviewed teachers reported low levels of comfort in teaching SEL, but the mean scores for the different facets ranged between medium levels of comfort.

## Discussion

The paper aimed at examining teachers’ SEL familiarity, beliefs, training, and perceived school culture by applying a mixed-methods approach. The results revealed that secondary school teachers reported to feel uncertain and lack the professional skills and knowledge to deliver SEL instructions. In fact, it was hard for teachers in the present study to define or describe the meaning of SEL and its facets. However, in line with Triliva and Poulou (2006), they did find themselves easier to define certain aspects of social awareness as an orientation toward others than defining the aspects that relate more to the self. Quantitative results supported our hypothesis that teachers’ comfort for teaching SEL was lower for self-awareness and self-management compared to social awareness.

In accordance with our hypotheses, we found a gap between the quantitative and qualitative part as the quantitative data showed, in general, higher levels of comfort as one would expect based on qualitative results. It seems that when secondary school teachers are asked to elaborate more closely on their familiarity and confidence and to provide their own ideas, it is more difficult for them to give clear answers. However, in our study, this may have been an effect of teachers’ level of job experience, as our interviewees had less job experience (5 years) compared to teachers who participated in the survey (10 years). Hence, in the future, more mixed-methods approaches seem to be necessary and highly valuable in order to provide a broader view on and a deeper understanding of teachers’ familiarity and comfort.

In both study parts, teachers expressed strong interest in receiving professional SEL training. One reason might be that our teachers had not participated in a structured SEL program yet. However, previous studies investigating teachers with or without participating in SEL programs also showed comparable high interest and commitment in SEL training (Triliva and Poulou, 2006; Buchanan et al., 2009; Collie et al., 2011, 2012, 2015; Brackett et al., 2012; Jones and Bouffard, 2012; Schonert-Reichl and Zakrzewski, 2014; Esen-Aygun and Sahin-Taskin, 2017). Hence, in future studies, it seems worthwhile to investigate more closely the differences between teachers who feel insecure and unprepared because they have not been in touch with the topic and the ones who feel uncomfortable regardless of the support they received in an SEL program. For the different SEL facets, contrary to our hypothesis, no differences in teachers’ commitment in learning about SEL were found. All means were rather high, including the one for self-management. Hence, although self-management or self-regulated skills gain more and more policy, research, and practical attention, teachers in this study still expressed a high need for learning how to teach these competencies.

How teachers should be trained in delivering SEL instruction is not answered sufficiently yet (Kimber et al., 2013). However, in order to be able to guide SEL instruction effectively, teachers need to be trained not only in delivering this type of instruction, but they also need to be skillful in SEL themselves (Jennings and Greenberg, 2009; Poulou, 2017b). Developing high SEL skills themselves may be related to a higher awareness of the importance of SEL. Moreover, they may function as role models for their students (Jennings and Greenberg, 2009; Zinsser et al., 2014). In addition, teachers’ social and emotional skills may be associated with the development of supportive teacher-student relationships, more effective classroom management, more effective SEL implementation in the classroom, and, at the same time, to less stress and teacher burnout (cf. Jennings and Greenberg, 2009). Hence, the development of teachers’ own social and emotional skills may have beneficial effects for teachers and their students next to a training with a focus on teaching SEL.

On the environmental side, qualitative and quantitative results revealed that teachers reported to feel less supported by the school administration in their attempts to deliver SEL instructions—mainly because they experience their schools to prioritize academic learning and outcomes, which leaves little room for explicit SEL. This result is in accordance with the argumentation of Durlak et al. (2011). They stated that—even though schools are important in preparing healthy learners by promoting not only academic development but also SEL—they are not capable of covering all learning aspects due to the scarcity of resources and intense heaviness of expectations to strengthen academic performance (Durlak et al., 2011). According to our interview data, secondary schools do not provide resources (instruction materials, specific courses, or activities) or create conditions (training teachers, devoting teaching hours, increasing number of counselors at schools, receiving school administration support) that would promote SEL instruction. If so, teachers reported different extracurricular activities as learning opportunities to foster SEL. However, simply because extracurricular activities are not plain academic content, they do not necessarily allow to develop students’ SEL. In addition, schools seem to focus more directly on responding to students’ social and emotional needs by offering discussions or school counseling services instead of teaching students how to develop their own social and emotional skills.

Quantitative data revealed differences in the perceived support of the school culture between the three facets. Schools seem to be more supportive of teaching and learning social awareness skills compared to self-awareness or self-management skills. This might explain why teachers also felt more comfortable in teaching social awareness compared to self-awareness and self-management. Hence, although offering teacher training for all facets seems to be important, our differential analyses showed an even higher need for providing an environment and teacher training on how to focus on the emotional, cognitive, and behavioral aspects of the self as compared to social aspects. In sum, our results show that, in future research, it is necessary and worthwhile to differentiate between SEL facets.

Overall, to support teachers in teaching SEL, a broader framework appears to be needed. At a macro level, an important step to promote SEL may be to define specific educational policies and include SEL in national standards and school laws (cf. Oberle and Schonert-Reichl, 2017). This applies to pre-, elementary, and secondary schools. As our results showed, there were hardly any differences between secondary school teachers’ SEL familiarity, beliefs, training, and perceived school culture compared to studies focusing on preschool or elementary school teachers. Some countries, for instance, the United States or Turkey, have just started such initiatives (cf. Esen-Aygun and Sahin-Taskin, 2017; Collaborative for Academic Social and Emotional Learning [CASEL], 2020). However, little is known about the application of these strategies and how the intended, formally established criteria are implemented in current school policies and academic curricula. When an explicit framework would exist, curricula in teacher education training on how to develop and teach SEL could be developed. Qualified teachers seem to be a key factor for developing social and emotional competencies successfully. Thus, they need to possess the capabilities, motivation, and resources to put SEL into action. Hence, future research is asked to combine the micro- with a macro-level perspective. These efforts appear to be worthwhile, as fostering SEL may enhance countries’ economic growth and contribute to higher social cohesion in the world.

## Limitations and Future Directions

Our study highlights the importance of teachers’ SEL familiarity, beliefs, training, and perceived school culture for investigating opportunities and practices for SEL instruction at schools. The study’s strengths are its focus on exploring teachers’ own attempts, initiatives, and instructional practices to enhance students’ SEL, the differential examination of SEL facets, and the mixed-methods approach.

Nevertheless, the study has certain limitations. One limitation is the composition of our sample. Our goal was to collect a detailed description of the phenomenon (cf. Creswell and Plano Clark, 2018). Therefore, we included secondary school teachers from different countries, asking about their beliefs and instructional approaches outside of structured SEL programs. Respondents were recruited based on the described criteria but not based on whether the country, where they had taught, already provided SEL policies. However, the availability of a statewide or nationwide policy and a country’s cultural background may indeed influence teachers’ SEL familiarity, beliefs, training, and perceived school culture (cf. Oberle and Schonert-Reichl, 2017). Hence, future studies may compare more systematically teacher familiarity, beliefs, training, and perceived school culture between countries with and without established SEL policies.

In addition, we focused on the facets self-awareness, self-management, and social awareness, as we expected these to be less often addressed in teaching in secondary schools but did not include relationship skills and responsible decision-making. However, our interview results showed that teachers already had a broader view of social awareness in mind, including many aspects that, according to the theoretical framework, would be assigned to relationship skills (Yopp et al., 2017). Hence, future research examining the effects of different SEL facets would benefit from (a) including all facets and (b) describing the facets and their differences more precisely.

One probably important belief we did not target specifically in our study is the malleability of students’ social and emotional skills. Teachers need to adopt a growth mindset and believe that these skills can be taught through formal instruction at school (cf. Seaton, 2018). Only then, they will put effort into developing their qualifications and devote time to target SEL explicitly in their classrooms. Hence, future studies may additionally consider teachers’ mindsets.

Finally, the perceived school culture and instructional practices were assessed by teachers only. Additional principal and student interviews would be a valuable source for getting more insights into their perspective of SEL instruction practices and school culture. Prior research on perceived teaching practices showed that students’ and teachers’ perceptions may differ and that sometimes rather students’ perception of teaching practices influences their learning (cf. Fauth et al., 2020).

## Conclusion

The present study adds to the literature on investigating teachers’ SEL familiarity and beliefs, their current SEL teaching practices, and the school culture in relation to SEL instruction where it takes place first—before the conduction of SEL programs and interventions. Our study results indicate that teachers’ familiarity with and their comfort in SEL teaching practices need to be strengthened. This could be achieved through providing support at two levels. At the micro level, pre-service and in-service teachers may benefit from professional education and training in developing their own SEL skills as well as on how to incorporate these topics in their regular teaching. At the macro level, SEL may need to be institutionalized on a policy level as it has already been done, for example, in some of the states in the United States, United Kingdom, or Turkey. By addressing both levels, teachers and schools would be better able to foster reaching multidimensional educational goals that include cognitive and non-cognitive outcomes.

## Data Availability Statement

The transcribed interviews and the dataset generated for the quantitative part of the study are available on request to the corresponding author. A full list of constructs assessed in this study can be found in the Appendices. So far, no other publications using these data are available.

## Ethics Statement

The study was conducted according to the Ethical Principles of Psychologists and Code of Conduct of the American Psychological Association from 2019. An ethics approval was not required by institutional guidelines or national regulations in line with the “German Research Foundation” guidelines as the used data was anonymized and no disclosure outside the research is possible. Participants were informed about the goals of the study, that participation was voluntarily, and that all data was confidential and would be used only in the frames of this research. For the qualitative part, interviews were recorded with respondents’ permission asked at the beginning of each interview.

## Author Contributions

AS-T introduced the articles’ idea, planned the article, and wrote most parts of the manuscript with contributions from AD and LZ. AS-T analyzed the quantitative data with support by LZ. AD’s master thesis built the foundation for the article and was supervised by AS-T. AD conducted the interviews and the online survey as well as analyzed and reported the qualitative part. All authors read and approved the final manuscript.

## Conflict of Interest

The authors declare that the research was conducted in the absence of any commercial or financial relationships that could be construed as a potential conflict of interest.

## Footnotes

1 https://www.google.com/forms/

## References

[B1] Aber J. L. Jones S. M. Brown J. L. Chaudry N. Samples F. (1998). Resolving conflict creatively: evaluating the developmental effects of a school-based violence prevention program in neighborhood and classroom context. *Dev. Psychopathol.* 10 187–213. 10.1017/s0954579498001576 9635221

[B2] American Psychological Association (2019). *Publication Manual of the American Psychological Association*, 7th Edn. Washington, DC: American Psychological Association.

[B3] Beland K. (2007). Boosting social and emotional competence. *Educ. Leadersh.* 64 68–71.

[B4] Blakemore S. Mills K. L. (2014). Is adolescence a sensitive period for sociocultural processing? *Ann. Rev. Psychol.* 65 187–207. 10.1146/annurev-psych-010213-115202 24016274

[B5] Bowden R. G. Lanning B. A. Pippin G. R. Tanner J. F. (2003). Teachers’ attitudes towards abstinence only sex education curricula. *Education* 123 780–790.

[B6] Boxer P. Guerra N. G. Huesmann L. R. Morales J. (2005). Proximal peer-level effects of a small-group selected prevention on aggression in elementary school children: an investigation of the peer contagion hypothesis. *J. Abnorm. Child Psychol.* 33 325–338. 10.1007/s10802-005-3568-2 15957560

[B7] Brackett M. A. Reyes M. R. Rivers S. E. Elbertson N. A. Salovey P. (2012). Assessing teachers’ beliefs about social and emotional learning. *J. Psychoeduc. Assess.* 30 219–236. 10.1177/0734282911424879

[B8] Brackett M. A. Rivers S. E. (2014). “Transforming students’ lives with social and emotional learning,” in *Educational Psychology Handbook Series. International Handbook of Emotions in Education*, eds Pekrun R. Linnenbrink-Garcia L. (Milton Park: Routledge), 368–388. 10.4324/9780203148211.ch19

[B9] Buchanan R. Gueldner B. A. Tran O. K. Merrell K. W. (2009). Social and emotional learning in classrooms: a survey of teachers’ knowledge, perceptions, and practices. *J. Appl. School Psychol.* 25 187–203. 10.1080/15377900802487078

[B10] Cole F. L. (1988). Content analysis: process and application. *Clin. Nurse Special. CNS* 2 53–57. 10.1097/00002800-198800210-00025 3349413

[B11] Collaborative for Academic Social and Emotional Learning [CASEL] (2020). *Collaborating States Initiative.* Chicago, IL: CASEL.

[B12] Collie R. J. Shapka J. D. Perry N. E. (2011). Predicting teacher commitment: the impact of school climate and social-emotional learning. *Psychol. Schools* 48 1034–1048. 10.1002/pits.20611

[B13] Collie R. J. Shapka J. D. Perry N. E. (2012). School climate and social–emotional learning: predicting teacher stress, job satisfaction, and teaching efficacy. *J. Educ. Psychol.* 104 1189–1204. 10.1037/a0029356

[B14] Collie R. J. Shapka J. D. Perry N. E. Martin A. J. (2015). Teachers’ beliefs about social-emotional learning: identifying teacher profiles and their relations with job stress and satisfaction. *Learn. Instruct.* 39 148–157. 10.1016/j.learninstruc.2015.06.002

[B15] Corcoran R. P. Cheung A. C. K. Kim E. Xie C. (2018). Effective universal school-based social and emotional learning programs for improving academic achievement: a systematic review and meta-analysis of 50 years of research. *Educ. Res. Rev.* 25 56–72. 10.1016/j.edurev.2017.12.001

[B16] Creswell J. W. Plano Clark V. L. (2018). *Designing and Conducting Mixed Methods Research*, 3rd Edn. Thousand Oaks, CA: Sage.

[B17] Denham S. A. Brown C. (2010). “Plays nice with others”: social–emotional learning and academic success. *Early Educ. Dev.* 21 652–680. 10.1080/10409289.2010.497450

[B18] Durlak J. A. (2016). Programme implementation in social and emotional learning: basic issues and research findings. *Cambridge J. Educ.* 46 333–345. 10.1080/0305764X.2016.1142504

[B19] Durlak J. A. Weissberg R. P. Dymnicki A. B. Taylor R. D. Schellinger K. B. (2011). The impact of enhancing students’ social and emotional learning: a meta-analysis of school-based universal interventions. *Child Dev.* 82 405–432. 10.1111/j.1467-8624.2010.01564.x 21291449

[B20] Durlak J. A. Weissberg R. P. Pachan M. (2010). A meta-analysis of after-school programs that seek to promote personal and social skills in children and adolescents. *Am. J. Community Psychol.* 45 294–309. 10.1007/s10464-010-9300-6 20300825

[B21] Dusenbury L. Zadrazil J. Mart A. Weissberg R. (2011). *State Learning Standards to Advance Social and Emotional Learning: The State Scan of Social and Emotional Learning Standards, Preschool Through High School.* Chicago, IL: Collaborative for Academic, Social, and Emotional Learning.

[B22] Eklund K. Kilpatrick K. D. Kilgus S. P. Haider A. (2018). A systematic review of state-level social–emotional learning standards: implications for practice and research. *School Psychol. Rev.* 47 316–326. 10.17105/SPR-2017.0116.V47-3

[B23] Elias M. J. Arnold H. (2006). *The Educator’s Guide to Emotional Intelligence and Academic Achievement: Social-Emotional Learning in the Classroom.* Thousand Oaks, CA: Corwin press.

[B24] Elias M. J. Zins J. E. Weissberg R. P. Frey K. S. Greenberg M. T. Haynes N. M. (1997). *Promoting Social and Emotional Learning: Guidelines for Educators.* Alexandria, VA: Association for Supervision and Curriculum Development.

[B25] Elo S. Kyngäs H. (2008). The qualitative content analysis process. *J. Adv. Nurs.* 62 107–115. 10.1111/j.1365-2648.2007.04569.x 18352969

[B26] Esen-Aygun H. Sahin-Taskin C. (2017). Teachers’ views of social-emotional skills and their perspectives on social-emotional learning programs. *J. Educ. Practices* 8 205–215.

[B27] Etikan I. Sulaiman A. M. Rukayya S. A. (2016). Comparison of convenience sampling and purposive sampling. *Am. J. Theoret. Appl. Statist.* 5 1–4. 10.11648/j.ajtas.20160501.11

[B28] Fauth B. Göllner R. Lenske G. Praetorius A.-K. Wagner W. (2020). Who sees what? conceptual considerations on the measurement of teaching quality from different perspectives. *Zeitschrift für Pädagogik* 66 138–155.

[B29] Field A. (2009). *Discovering Statistics Using SPSS.* Thousand Oaks, CA: Sage.

[B30] Fives H. Buehl M. M. (2012). “Spring cleaning for the “Messy” construct of teachers’ beliefs: what are they? which have been examined? what can they tell us?,” in *APA Handbooks in Psychology. APA Educational Psychology Handbook*, 1st Edn, eds Graham S. Urdan T. (Washington, DC: American Psychological Association), 471–499.

[B31] Gebauer M. M. McElvany N. (2017). Empirische Arbeit: zur Bedeutsamkeit unterrichtsbezogener heterogenitätsspezifischer Einstellungen angehender Lehrkräfte für intendiertes Unterrichtsverhalten [Impact of prospective teachers’ heterogeneity specific attitudes on intended teaching behavior]. *Psychol. Erziehung Unterricht* 64:163. 10.2378/peu2017.art11d

[B32] Gingiss P. L. Gottlieb N. H. Brink S. G. (1994). Increasing teacher receptivity toward use of tobacco prevention education programs. *J. Drug Educ.* 24 163–176. 7931926 10.2190/2UXC-NA52-CAL0-G9RJ

[B33] Google (2020). *Google ist der Einhaltung der Anwendbaren Datenschutzgesetze Verpflichtet [Google is committed to compliance with applicable privacy laws].* Mountain View, CA: Google.

[B34] Graczyk P. A. Domitrovich C. E. Small M. Zins J. E. (2006). Serving all children: an implementation model framework. *School Psychol. Rev.* 35 266–274. 10.1080/02796015.2006.12087991

[B35] Greenberg M. T. Weissberg R. P. O’Brien M. U. Zins J. E. Fredericks L. Resnik H. (2003). Enhancing school-based prevention and youth development through coordinated social, emotional, and academic learning. *Am. Psychol.* 58 466–474. 10.1037/0003-066x.58.6-7.466 12971193

[B36] Harrell A. W. Mercer S. H. DeRosier M. E. (2009). Improving the social-behavioral adjustment of adolescents: the effectiveness of a social skills group intervention. *J. Child Family Stud.* 18 378–387. 10.1007/s10826-008-9241-y

[B37] Heyvaert M. Hannes K. Maes B. Onghena P. (2013). Critical appraisal of mixed methods studies. *J. Mixed Methods Res.* 7 302–327. 10.1177/1558689813479449

[B38] Jennings P. A. Greenberg M. T. (2009). The prosocial classroom: teacher social and emotional competence in relation to student and classroom outcomes. *Rev. Educ. Res.* 79 491–525. 10.3102/0034654308325693

[B39] Jones S. M. Bouffard S. M. (2012). Social and emotional learning in schools: from programs to strategies and commentaries. *Soc. Policy Report* 26 1–33. 10.1002/j.2379-3988.2012.tb00073.x

[B40] Kautz T. Heckman J. Diris R. ter Weel B. Borghans L. (2014). *Fostering and Measuring Skills: Improving Cognitive and Non-Cognitive Skills to Promote Lifetime Success.* Cambridge, MA: OECD Publishing, 10.3386/w20749

[B41] Kimber B. Skoog T. Sandell R. (2013). Teacher change and development during training in social and emotional learning programs in Sweden. *Int. J. Emot. Educ.* 5 17–35.

[B42] Liew J. McTigue E. M. (2010). “Educating the whole child: the role of social and emotional development in achievement and school success,” in *Education in a Competitive and Globalizing World. Handbook of Curriculum Development*, ed. Kattington L. E. (Hauppauge, NY: Nova Science), 465–478.

[B43] Mahoney J. L. Durlak J. A. Weissberg R. P. (2018). An update on social and emotional learning outcome research. *Phi Delta Kappan* 100 18–23. 10.1177/0031721718815668

[B44] Metropolitan Area, Child Study, and Research Group. (2002). A cognitive-ecological approach to preventing aggression in urban settings: initial outcomes for high-risk children. *J. Consult. Clin. Psychol.* 70 179–194. 11860044

[B45] Oberle E. Schonert-Reichl K. A. (2017). “Social and emotional learning: recent research and practical strategies for promoting children’s social and emotional competence in schools,” in *Handbook of Social Behavior and Skills in Children*, ed. Matson J. L. (Berlin: Springer), 175–197.

[B46] OECD (2015). *OECD Skills Studies: Skills for Social Progress: The Power of Social and Emotional Skills.* Paris: OECD Publishing.

[B47] Pajares M. F. (1992). Teachers’ beliefs and educational research: cleaning up a messy construct. *Rev. Educ. Res.* 62 307–332. 10.2307/1170741

[B48] Parcel G. S. O’Hara-Tompkins N. M. Harrist R. B. Basen-Engquist K. M. (1995). Diffusion of an effective tobacco prevention program: II. evaluation of the adoption phase. *Health Educ. Res.* 10 297–307. 10.1093/her/10.3.297 10158027

[B49] Poulou M. S. (2017a). An examination of the relationship among teachers’ perceptions of social-emotional learning, teaching efficacy, teacher-student interactions, and students’ behavioral difficulties. *Int. J. School Educ. Psychol.* 5 126–136. 10.1080/21683603.2016.1203851

[B50] Poulou M. S. (2017b). The relation of teachers’ emotional intelligence and students’ social skills to students’ emotional and behavioral difficulties: a study of preschool teachers’ perceptions. *Early Educ. Dev.* 28 996–1010. 10.1080/10409289.2017.1320890

[B51] Rieger S. Göllner R. Spengler M. Trautwein U. Nagengast B. Roberts B. W. (2017). Social cognitive constructs are just as stable as the big five between grades 5 and 8. *AERA Open* 3 1–9. 10.1177/2332858417717691

[B52] Schonert-Reichl K. Zakrzewski V. (2014). *How to Close the Social-Emotional Gap in Teacher Training.* Berkeley, CA: Greater Good Magazine.

[B53] Schonert-Reichl K. A. Hanson-Peterson Jennifer L. Hymel Shelley. (2015). “SEL and preservice teacher education,” in *Handbook of Social and Emotional Learning: Research and Practice*, eds Durlak J. A. Celene D. Weissberg R. P. Gullotta T. P. (New York, NY: Guilford), 406–421.

[B54] Schonert-Reichl K. A. Kitil M. J. Hanson-Peterson J. (2016). *Teachers First: A National Scan of Teacher Preparation Programs and Social and Emotional Learning. A Report Prepared for the Collaborative for Academic, Social, and Emotional Learning (CASEL).* Vancouver, BC: University of British Columbia.

[B55] Schunk D. H. Zimmerman B. J. (2012). *Motivation and Self-regulated Learning: Theory, Research, and Applications.* Milton Park: Routledge.

[B56] Seaton F. S. (2018). Empowering teachers to implement a growth mindset. *Educ. Psychol. Pract.* 34 41–57. 10.1080/02667363.2017.1382333

[B57] Skaalvik E. M. Skaalvik S. (2011). Teacher job satisfaction and motivation to leave the teaching profession: relations with school context, feeling of belonging, and emotional exhaustion. *Teach. Teacher Educ.* 27 1029–1038. 10.1016/j.tate.2011.04.001

[B58] Sklad M. Diekstra R. Ritter M. D. E. Ben J. Gravesteijn C. (2012). Effectiveness of school-based universal social, emotional, and behavioral programs: do they enhance students’ development in the area of skill, behavior, and adjustment? *Psychol. Schools* 49 892–909. 10.1002/pits.21641

[B59] Taylor R. D. Oberle E. Durlak J. A. Weissberg R. P. (2017). Promoting positive youth development through school-based social and emotional learning interventions: a meta-analysis of follow-up effects. *Child Dev.* 88 1156–1171. 10.1111/cdev.12864 28685826

[B60] Tolan P. H. Guerra N. G. Kendall P. C. (1995). A developmental-ecological perspective on antisocial behavior in children and adolescents: toward a unified risk and intervention framework. *J. Consult. Clin. Psychol.* 63 579–584. 10.1037//0022-006X.63.4.579 7673535

[B61] Triliva S. Poulou M. (2006). Greek teachers’ understandings and constructions of what constitutes social and emotional learning. *School Psychol. Int.* 27 315–338. 10.1177/0143034306067303

[B62] Trivette C. M. Dunst C. J. Hamby D. W. Meter D. (2012). Relationship between early childhood practitioner beliefs and the adoption of innovative and recommended practices. *Res. Brief* 6 1–12.

[B63] Wanless S. B. Patton C. L. Rimm-Kaufman S. E. Deutsch N. L. (2013). Setting-level influences on implementation of the responsive classroom approach. *Prevent. Sci.* 14 40–51. 10.1007/s11121-012-0294-1 23065349

[B64] Wigelsworth M. Lendrum A. Oldfield J. Scott A. ten Bokkel I. Tate K. (2016). The impact of trial stage, developer involvement and international transferability on universal social and emotional learning programme outcomes: a meta-analysis. *Cambridge J. Educ.* 46 347–376. 10.1080/0305764X.2016.1195791

[B65] Yeager D. S. (2017). Social and emotional learning programs for adolescents. *Future Child* 27 73–94. 10.1353/foc.2017.0004

[B66] Yopp A. McKimm B. Moore L. Odom S. Hanagriff R. (2017). A multidimensional needs assessment of social emotional learning skill areas. *J. Agricul. Educ.* 58 186–206. 10.5032/jae.2017.01186

[B67] Zins J. E. Elias M. J. (2007). Social and emotional learning: promoting the development of all students. *J. Educ. Psychol. Consult.* 17 233–255. 10.1080/10474410701413152

[B68] Zins J. E. Weissberg R. P. Wang M. C. Walberg H. J. (2004). *Building Academic Success on Social and Emotional Learning: What Does the Research Say?*, Ed Edn. New York, NY: Teachers College Press.

[B69] Zinsser K. M. Shewark E. A. Denham S. A. Curby T. W. (2014). A mixed-method examination of preschool teacher beliefs about social–emotional learning and relations to observed emotional support. *Infant Child Dev.* 23 471–493.

