# Supplementary material for: A Mixed-Methods Approach to Investigating Social and Emotional Learning at Schools: Teachers’ Familiarity, Beliefs, Training, and Perceived School Culture
Source: Front Psychol. 2021 Jun 1;12:518634. doi: 10.3389/fpsyg.2021.518634 (PMC8204053; doi:10.3389/fpsyg.2021.518634)
Supplement: Supplementary file 1 [file Data_Sheet_1.pdf]

Appendix A

Description of Interview Participants

#	Age	Gender	Country	Subjects taught	Grade	Years of Experience	Type of school
Teacher #1	24	Female	Kyrgyzstan	Sociology, English, History	9th – 12th	3	Private
Teacher #2	24	Male	Kyrgyzstan	English	3rd-10th	3	Private + Public
Teacher #3	25	Female	Kyrgyzstan	Geography	6th- 11th	2,5	Public
Teacher #4	50	Female	Kyrgyzstan	Math	5th-11th	19	Public
Teacher #5	31	Male	USA	History, English, Psychology	7th-11th	5,5	Public
Teacher #6	24	Female	China	English	4th -6th	4	Public
Teacher #7	25	Female	Germany	German, business administration and nutrition science	9th and 10th	2	Public
Teacher #8	26	Female	Brazil	Preschool teacher	pre-school	5	Private
Teacher #9	28	Female	USA	Math and Algebra	9th-12th	5	Public
Teacher #10	25	Female	Turkey	Math	6th – 11th	5	Public + Private
Teacher #11	24	Female	Turkey	English	9th – 10th	2	Private
Teacher #12	28	Male	USA	English and Literature	7th – 12th	5	Public
Teacher #13	24	Female	Finland	English, Crafts	6th – 9th	3	Public
Teacher #14	27	Female	South Korea	English	6th – 9th	5	Public
