# Supplementary material for: A Mixed-Methods Approach to Investigating Social and Emotional Learning at Schools: Teachers’ Familiarity, Beliefs, Training, and Perceived School Culture
Source: Front Psychol. 2021 Jun 1;12:518634. doi: 10.3389/fpsyg.2021.518634 (PMC8204053; doi:10.3389/fpsyg.2021.518634)
Supplement: Supplementary file 2 [file Data_Sheet_2.pdf]

Appendix B

Interview Guide

Hello Dear___,
Thank you for your time and participation in this study. As you know this study aims at exploring how teachers and schools support students' social- emotional learning in terms of students' self-awareness, self-management and social-awareness skills. I am interested in your experiences and opinions regarding this topic. There are no right or wrong answers, I value any information you would consider sharing with us. If you don't mind, I would like to record this interview in order to use the data later for data analysis and I assure you that the information you provide during the interview will be absolutely confidential and will be used only in the frames of this research. The interview might be 30-40 mins long and you have an absolute right to stop it at any time you need or skip the questions you feel not comfortable answering. Thank you once again and if you have no objections, we can start.

1. Introduction and definition:

a) Could you please introduce yourself by telling where you come from, where you live now and how old you are?
How long have you worked as a teacher?
Which subjects have you taught?
Which grades have you taught?
In which type of school have you been working?
Thank you!

b) Since the focus of my study is on SEL and its three facets self- awareness, self-management, and social-awareness, I was curious about how you would personally define the terms of 1) SEL, 2) self-awareness, 3) self-management and 4) social-awareness?

c) Thank you before we continue, I would like to mention how this study defines these competencies:

Social emotional learning involves processes of combining thinking, feeling, and behaving in order to become aware of the self and of others, to regulate self-behavior and those of others and to make responsible decisions.
Self-awareness is the ability to carefully identify one's own emotions, thoughts, interests, and values and understand how they impact behavior as well as the ability to accurately evaluate one's strengths and limitations and maintain a well-grounded sense of self-efficacy and self-confidence.
Self-management involves skills such as self-discipline, motivation, goal setting and stress management. It is the ability to regulate one's emotions, thoughts, and behaviors in various situations and being able to set and monitor progress towards personal and academic aims.
Social-awareness is the ability to have respect and empathy for others and understand others' perspectives and feelings. It is also an ability to perceive similarities and differences among people.

2. School support
   a) With these definitions in mind, do you think your school provides opportunities for developing self-awareness, self-management and social-awareness in students? If yes, how? If now, why?
   b) From your experience, which content does the school curriculum provide in relation to students’ self-awareness, self-management and social-awareness ?
   c) Are there any materials that cover content related to students’ self-awareness, self-management, and social-awareness?
   d) Are there any extracurricular activities (study courses, sports, art classes and so on) that aim at developing students’ self-awareness, self-management, and social-awareness competencies ?
   e) Does your principal encourage the teaching of self-awareness, self-management, and social-awareness competencies?
   f) Does your school expect teachers to address students' self-awareness, self-management, and social-awareness needs?
   g) Are there any consulting services that your school provides to support students interests or concerns? (like advising places for graduating students or psychological counselors or social workers)

3. Teachers’ comfort with and individual support of SEL
   a) What do you think, can you as a teacher have an impact on students’ self-awareness, self- management, and social-awareness skills? If yes, in what ways? If no, why do you think so?
   b) Do you feel confident and comfortable in your ability to provide instruction on students’ self-awareness, self-management, and social-awareness learning?
   c) Do you, as a teacher, have enough opportunities (like time, study plan, size of the class) to recognize and develop students’ self-awareness, self-management, and social- awareness?
   d) What are the challenges that you, as a teacher, can face while working with students’ on self-awareness, self-management, and social-awareness?

4. Teacher training
   a) During your studies, did you have any courses on the topic of students’ self- awareness, self-management, or social-awareness?
   b) Have you ever attended a workshop, training, or conference related students’ self-awareness, self-management, or social-awareness competencies?
   c) As a teacher, what would you recommend that schools do in order to foster students’ personality development?

Thank you very much for such an interesting interview! We appreciate your time and interest in our study. If you have anything else to share, comment, provide feedback or ask, please feel free to do so.
