# Supplementary material for: A Mixed-Methods Approach to Investigating Social and Emotional Learning at Schools: Teachers’ Familiarity, Beliefs, Training, and Perceived School Culture
Source: Front Psychol. 2021 Jun 1;12:518634. doi: 10.3389/fpsyg.2021.518634 (PMC8204053; doi:10.3389/fpsyg.2021.518634)
Supplement: Supplementary file 3 [file Data_Sheet_3.pdf]

Appendix C

Teachers' SEL Beliefs Scale adapted from Brackett et al. (2012) and demographic questions

Self-awareness

Self-awareness is the ability to carefully identify one's own emotions, thoughts, interests, and values and understand how they impact one's behavior (Eklund, Kilpatrick, Kilgus & Haider, 2018). It is the ability to accurately evaluate one's strengths and limitations and maintain a well-grounded sense of self-efficacy and self-confidence (Brackett & Rivers, 2014, Eklund, Kilpatrick, Kilgus & Haider, 2018; Zins & Elias, 2007; Denham & Brown, 2010).

With this definition in mind, please read the following statements and think about how true each is for YOU. Rate the extent to which you agree or disagree with each statement.

1. I feel confident in my ability to provide instruction on students' sense of self-awareness.
2. I am comfortable providing instruction on self-awareness skills to my students.
3. Taking care of my students' self-awareness needs comes naturally to me.
4. Informal lessons about self-awareness are part of my regular teaching practice.
5. I would like to attend a workshop to learn how to develop my students' self-awareness skills.
6. I would like to attend a workshop to develop my own self-awareness skills.
7. I want to improve my ability to teach self-awareness skills to students.
8. All teachers should receive training on how to teach self-awareness skills to students.
9. My principal creates an environment that promotes self-awareness learning for our students.
10. The culture in my school supports the development of students' self-awareness skills.
11. My principal does not encourage the teaching of self-awareness skills to students.
12. My school expects teachers to address students' self-awareness needs.

Self-management

Self-management involves skills such as self-discipline, motivation, goal setting, and stress management (Dusenbury, Zadrazil, Mart & Weissberg, 2011). It is the ability to regulate one’s emotions, thoughts, and behaviors in various situations and being able to set and monitor progress towards personal and academic aims (Brackett & Rivers, 2014; Eklund, Kilpatrick, Kilgus & Haider, 2018).

With this definition in mind, please read the following statements and think about how true each is for YOU. Rate the extent to which you agree or disagree with each statement.

1. I feel confident in my ability to provide instruction on students’ self-management.
2. I am comfortable providing instruction on self-management skills to my students.
3. Taking care of my students’ self-management needs comes naturally to me.
4. Informal lessons in self-management are part of my regular teaching practice.
5. I would like to attend a workshop to learn how to develop my students’ self-management skills.
6. I would like to attend a workshop to develop my own self-management skills.
7. I want to improve my ability to teach self-management skills to students.
8. All teachers should receive training on how to teach self-management skills to students.
9. My principal creates an environment that promotes self-management learning for our students.
10. The culture in my school supports the development of students' self-management skills.
11. My principal does not encourage the teaching of self-management skills to students.
12. My school expects teachers to address students' self-management needs.

Social awareness

Social awareness is the ability to have respect and empathy for others and understand others’ perspectives and feelings (Denham & Brown, 2010, Zins & Elias, 2007). It is also the ability to perceive similarities and differences among people (Denham & Brown, 2010).

With this definition in mind, please read the following statements and think about how true each is for YOU. Rate the extent to which you agree or disagree with each statement.

1. I feel confident in my ability to provide instruction on students’ sense of social awareness.
2. I am comfortable providing instruction on social awareness skills to my students.
3. Taking care of my students’ social awareness needs comes naturally to me.
4. Informal lessons in social awareness are part of my regular teaching practice.
5. I would like to attend a workshop to learn how to develop my students’ social awareness skills.
6. I would like to attend a workshop to develop my own social awareness skills.
7. I want to improve my ability to teach social awareness skills to students.
8. All teachers should receive training on how to teach social awareness skills to students.
9. My principal creates an environment that promotes social awareness learning for our students.
10. The culture in my school supports the development of students’ social awareness skills.
11. My principal does not encourage the teaching of social awareness skills to students.
12. My school expects teachers to address students’ social awareness needs.

Demographic questions

1. What is your age?
2. What is your gender?
3. Where are you from (country)?
4. Where do you currently live (country)?
5. How long have you worked as a teacher?
6. Which subjects have you taught?
7. Which grades have you taught?
8. In which type of school have you been working?
